# Chemical Composition, Antimicrobial Activity, and Withdrawal Period of Essential Oil-Based Pharmaceutical Formulation in Bovine Mastitis Treatment

**DOI:** 10.3390/ijerph192416643

**Published:** 2022-12-11

**Authors:** Zorana Kovačević, Dragana Tomanić, Ivana Čabarkapa, Ljubiša Šarić, Jovan Stanojević, Katarina Bijelić, Ivan Galić, Zoran Ružić, Mihajlo Erdeljan, Nebojša Kladar

**Affiliations:** 1 Department of Veterinary Medicine, Faculty of Agriculture, University of Novi Sad, 21000 Novi Sad, Serbia; 2 Institute of Food Technology, University of Novi Sad, 21000 Novi Sad, Serbia; 3 Center for Medical and Pharmaceutical Investigations and Quality Control, Department of Pharmacy, Faculty of Medicine, University of Novi Sad, 21000 Novi Sad, Serbia

**Keywords:** essential oils, withdrawal period, mastitis, antimicrobials, Phyto-Bomat, residues

## Abstract

Due to the emergence of antibiotic-resistant bacteria, the risk it represents to public health, and the possible consequences for animal health and welfare, there is an increasing focus on reducing antimicrobial usage (AMU) in animal husbandry. Therefore, a great interest in developing alternatives to AMU in livestock production is present worldwide. Recently, essential oils (EOs) have gained great attention as promising possibilities for the replacement of antibiotics. The current study aimed to test the potential of using a novel EO-based pharmaceutical formulation (Phyto-Bomat) in bovine mastitis treatment. The antibacterial activity was performed using the microdilution technique. Lactating dairy cows were treated with 15 mL of Phyto-Bomat in the inflamed quarter for 5 consecutive days in order to analyze blood and milk samples for thymol and carvacrol residues using gas chromatography and mass spectrometry (GC–MS). Antimicrobial activity expressed as the minimum inhibitory concentration (MIC) and minimal bactericidal concentration (MBC) indicates that this formulation has the highest activity against Gram-positive strains. The dominant compounds in Phyto-Bomat were thymol and carvacrol, at 12.58 ± 1.23 mg/mL and 23.11 ± 2.31 mg/mL, respectively. The quantification of these two compounds in evaluated biological samples showed that 24 h after administration the concentration of thymol and carvacrol in milk samples was at the same level as before application. On the other hand, thymol and carvacrol were detectable in plasma samples even after 24 h post-treatment, with values ranging from 0.15–0.38 and 0.21–0.66 µg/mL, respectively. The tested formulation showed encouraging results of antibacterial activity against bovine mastitis pathogens, as well as the withdrawal period of dominant compounds, which implies that further testing regarding the bacteriological and clinical cure rates in clinical settings is needed.

## 1. Introduction

Bovine mastitis is among the most severe and economically important infections affecting livestock production, and one of the major causes of antibiotic use in dairy cows [1,2]. In general, mastitis is defined as an inflammation of the mammary gland, usually caused by different pathogenic microorganisms, mostly bacteria, such as staphylococci, streptococci, and coliforms [1,3]. Moreover, *Staphylococcus aureus*, *Streptococcus uberis*, *Escherichia coli*, and *Streptococcus agalactiae* are among the most frequent mastitis-associated pathogens in Serbia [4], but in other European countries also [1,5,6].

Apart from the substantial economic losses associated with the disease, it has extreme zoonotic importance since the milk is unsafe for human consumption [7,8]. This unsafety could be due to the presence of residues and the long withdrawal period of antimicrobials [9,10]. Moreover, antimicrobial residues in milk can interfere with the production of dairy products and may cause hypersensitivity and resistance to microorganisms in humans [11]. Furthermore, the increasing concern about antibiotic resistance in public health issues is pushing the milk industries to reduce the usage of antimicrobial drugs [9]. Erskine et al. [12] reported that approximately 90% of the residues detected in milk over a period of five years originated from antibacterial therapy for mastitis.

Therefore, there is a growing need to develop new, alternative therapies, especially those derived from natural products, such as plants [8,13]. That is one reason why phytotherapy is gaining much attention nowadays as an alternative to antimicrobial agents. Considering the numerous advantages that essential oils (EOs) have in relation to antibiotics—such as non-toxicity, biodegradability, and reduced possibility of resistance—in recent decades, their research and use have been gaining attention [14]. In addition to the mentioned advantages of phytotherapy, in recent years, there has been a large decline in the percentage of newly discovered antibiotics, which could be an alternative to the existing ones, whose efficiency is decreasing [15].

EOs are aromatic oily liquids obtained from different plant parts and are widely used in several industrial and scientific fields [16,17]. Many of them have the ‘generally recognized as safe’ (GRAS) status, awarded by the United States Food and Drug Authority (FDA) [18]. According to traditional medicinal knowledge, EOs have been used as analgesics, sedatives, anxiolytics, antifungals, anti-inflammatory drugs, and antibacterial agents [19]. EOs have been recognized for their potential antimicrobial activities due to their high hydrophobicity, which enables them to cross the bacterial cell membranes leading to a loss of function and damage of proteins, lipids, and organelles within the bacterial cell, and consequently cell death [20,21,22].

*Thymus vulgaris* L., *Thymus serpyllum* L., *Satureja montana* L., and *Origanum vulgare* L. belong to the *Lamiaceae* family, known for its diverse biological and pharmacological properties [23]. EOs from the genus *Thymus* exhibit different biological properties such as antioxidant, antibacterial, antifungal, antiviral, antiparasitic, cytotoxic, and spasmolytic [24,25]. In addition, EOs from various *Satureja* species have also demonstrated antibacterial, antiviral, antiparasitic, antioxidant, anti-inflammatory, carminative, and digestive properties [26,27]. Moreover, EOs of oregano species are widely recognized for their antimicrobial, anti-inflammatory, immunostimulating, antioxidant, spasmolytic, analgesic, anxiolytic, antimutagenic, and antigenotoxic effects [28,29].

Additionally, there is potential to decrease antimicrobial consumption and consequently antimicrobial resistance through the development of EO-based phytopharmaceuticals for mastitis treatment due to the shorter withdrawal period of EOs. Actually, mastitis in lactating cows is commonly treated by intramammary or parenterally infusion of antibiotics [8,30]. Previous research suggests that the most commonly used antibiotics in mastitis therapy in Serbia were penicillin, streptomycin, gentamicin, tetracycline, cephalexin, sulfonamides, and enrofloxacin [31,32]. According to the Summary of the Product characteristics of these drugs given by the Medicines and Medical Devices Agency of Serbia, the withdrawal period of these antibiotics can vary from 1–5 days, while McPhee et al. [33] reported that thymol residues were only detected in the 12 h post-treatment in the milk sample. However, some authors reported that the activity of some antibiotics such as macrolides, tetracyclines and trimethoprim-sulphonamides is reduced in milk, which also reduces the chances of effective treatment [10,14].

Hence, the aim of the present study was to evaluate the antimicrobial activity of an EO-based intramammary pharmaceutical formulation developed for bovine mastitis treatment. In addition, the withdrawal period of the proposed formulation in the milk and blood of treated cows was assessed.

## 2. Materials and Methods

### 2.1. EO-Based Formulation

The proposed pharmaceutical formulation for intramammary application was based on four different EOs with proven antimicrobial activity. Namely, it contained EOs of common (*Thymus vulgaris* L.) and wild thyme (*Thymus serpyllum* L.), oregano (*Origanum vulgare* L.), and mountain savory (*Satureja montana* L). The obtained EO mixture was further diluted with common marigold (*Calendula officinalis* L.) and St. John’s wort (*Hypericum perforatum* L.) oil macerates (herbal drug:sunflower oil, 1:5) in an amount of up to 15 mL in an intramammary injector. The chemical composition and antimicrobial activity of common and wild thyme against bovine mastitis-associated pathogens were previously studied by Kovacevic et al. [4]. Oregano and mountain savory chemical compositions and antimicrobial activity against bovine mastitis-associated pathogens were reported by Kovacevic et al. [34]. The EOs’ concentration in the proposed formulation was determined according to the MBC values against the most common mastitis-associated pathogens. The predominant compounds among the EO components included in the proposed formulation were thymol and carvacrol [4,34].

### 2.2. Sampling Procedure

The experimental protocol was approved by the Animal Ethics Committee of the Ministry of Agriculture, Forestry and Water Management-Veterinary Directorate (9000-689/2, 7 June 2020). The presented study was carried out at two dairy farms located in Serbia, with 500–1100 Holstein-Friesian cows per farm. Milk samples were collected from individual quarters with clinical and subclinical mastitis. The cows were screened for clinical mastitis by clinical examination, while subclinical mastitis was assessed using somatic cell count in the milk samples. Palpation and inspection methods were performed to examine typical signs of clinical mastitis by a veterinarian. Pathogen isolation was conducted from October 2021 to December 2021 by taking milk samples from all animals during morning milking. A total of 55 milk samples from dairy cows at two farms, diagnosed with mastitis during the study period were sampled. Before sampling, the udder was cleaned and wiped. The tips of the teats and the openings of the suction canal were cleaned and disinfected with a cotton swab soaked in 70% alcohol. The first jets of milk were discarded, after which a few milliliters of milk were milked into sterile tubes. After the milk samples were collected, they were immediately transported to the Laboratory for Milk Hygiene at the Department of Veterinary Medicine, Faculty of Agriculture, University of Novi Sad, under the cold chain (4 °C). All milk samples were incubated on nutrient agar with the addition of 2% blood and incubated under aerobic conditions for 48 h at 37 °C, using a platinum loop (0.01 mL). Microorganisms were isolated and identified based on morphological and biochemical characteristics, as described by Kovacevic et al. [4].

### 2.3. EOs’ Effectiveness Determination against Mastitis-Associated Bacteria

The effectiveness of the solution of the final preparation on microorganisms was determined according to the Clinical Laboratory Standards [35] with slight modifications. Mueller–Hinton broth (MHB, HiMedia) was inoculated into each well of a microtiter plate (except for the first well) in a total volume of 100 µL. The first well of the microtiter plate was inoculated with 100 µL of pure preparation (909.09 µL/mL). The second well of the microtiter plate was inoculated with 100 µL of pure preparation and represented a stock solution that contained 100µL EO + 100 µL broth (454.54 µL/mL). Afterwards, serial doubling dilutions of the tested EOs were prepared in a 96-well microtiter plate well (Hillium) over a range of 454.54 to 56.81 µL/mL (Table 1). Finally, 100 µL was removed from the last well of the microtiter plate. Then, 10 µL of bacterial suspension was added to each test well. The final volume in each well was 110 µL/mL and the final bacterial concentration was 10^6^ CFU/mL. The plate was incubated for 24 h at 37 °C. The same tests were performed simultaneously for growth control (MHB + test organism), sterility control I (MHB +preparation), and sterility control II (MHB). The growth of microorganisms was determined by adding 10 µL at 0.01% of the resazurin solution (HiMedia). The plates were incubated at 37 °C for 24 h (in darkness). The change in color from blue (oxidized) to pink (reduced) indicated the growth of bacteria. The minimum inhibitory concentration (MIC) was determined as the lowest concentration of the final preparation that prevented the transition of oxidated to the reduced form of resazurin and was determined by cultivating 100 µL of solution from each well of the microtiter plate in Mueller–Hinton agar (MHA, HiMedia) [36]. The plates were incubated at 37 °C for 24 h. The minimum bactericidal concentration (MBC) was defined as the lowest concentration of the final preparation solution at which 99.9% of inoculated bacteria were killed.

### 2.4. Therapeutic/Experimental Protocol

EO-based intramammary formulation with previously suggested in vitro antimicrobial effect was tested in vivo on cows with mastitis. Animals with a positive diagnosis of mastitis (*n* = 55) were chosen in the present experiment for in vivo tests. Formulation was administered intramammarily, twice a day, after milking, in 15 mL volume, for 5 consecutive days. The formulation contained EOs of oregano, mountain savory, and common and wild thyme in different concentrations. The milk and blood samples were obtained before treatment, as well as 12 h and 24 h after the treatment. Blood samples were collected in citrate-containing vacutainers, centrifuged, and the obtained blood plasma was kept at −20 °C until analyzed. Milk samples were also kept at −20 °C until analysis.

### 2.5. Withdrawal Period of EO-Based Intramammary Formulation

#### 2.5.1. Method Development and Validation

Chemical standard substances of thymol and carvacrol (Sigma-Aldrich, St. Louis, MO, USA) were dissolved in acetonitrile to obtain stock standard solutions in a concentration of 100 µg/mL. Stock standard solutions were diluted with acetonitrile in order to obtain working standard solutions (10 µg/mL) which were used for preparing calibration standard solutions containing thymol and carvacrol in concentrations ranging from 0.067–6.67 µg/mL and ketamine hydrochloride (internal standard) in a concentration of 0.5 µg/mL. The prepared calibration solutions were analyzed via gas chromatography–mass spectrometry (GC–MS) instrumental technique (7890B GC System, 5997A MSD; Agilent Technologies, Waldbronn, Germany). The compounds of interest were separated on HP-5 ms (30 m) capillary column, where 1 µL of sample was injected in splitless mode at the inlet temperature of 260 °C. The starting oven temperature was 50 °C and held for 1 min after which the temperature was raised to 165 °C at a rate of 30 °C/min and held for 5 min. The second ramp was set at 195 °C at a rate of 9 °C/min and held for 10 min, while the third ramp was set at 280 °C at a rate of 40 °C/min and held for 7 min. The MSD transfer line was set at 280 °C, and the total run took 32 min. The obtained chromatograms were monitored in SCAN (*m*/*z*: 50–330) and SIM modes (*m*/*z*: 91, 135, 150, 180, 182, 209). The analytical method has been validated in terms of selectivity, linearity, precision (repeatability and reproducibility), accuracy, limits of detection (LOD), and quantification (LOQ). The selectivity of the method was assessed based on the chromatograms of the calibration solution containing thymol and carvacrol, as well as real samples. Linearity was estimated by least squares regression analysis of the results obtained for calibration curves (at 8 points, obtained in duplicates) ranging from 0.067–6.67 µg/mL. The intra- and inter-day (*n* = 3) precision were evaluated by analysis of independently prepared samples of different matrix types. Accuracy was determined by spiking real samples at three different concentration levels (0.5, 3, and 6 µg/mL). The LOD and LOQ were estimated by injecting previously spiked samples (0.1µg/mL), which naturally did not contain thymol and carvacrol.

#### 2.5.2. Preparation of Samples

After thawing, the appropriate amount of biological sample (3 mL of milk or 1 mL of blood plasma) was accurately measured in conical tube, saturated with ammonia sulfate, closed with a rubber stop, and vortexed for 3 min. After that, 1.5 mL of ketamine hydrochloride solution in diethyl ether (c = 0.5 µg/mL) was added to the tubes and vortexed for another 5 min. The samples were then centrifuged (10 min, 3900 rpm) and diethyl ether extracts were transferred to an evaporating dish for gentle removal of solvent in air stream. The dry residue was dissolved in 1.5 mL of acetonitrile and analyzed through a GC–MS instrument according to previously described conditions.

Phyto-Bomat preparation was extracted with ketamine solution in acetonitrile (c = 0.5 µg/mL) and analyzed via described GC–MS technique.

### 2.6. Data Analysis

All of the obtained data were analyzed with Microsoft Office Excel v 2019. And Statsoft Statistica v12.5. The results were processed by means of descriptive statistics, while differences between concentrations of quantified compounds (thymol and carvacrol) in relation to evaluated time points were assessed through application of ANOVA followed by post-hoc Tukey HSD test. The differences were considered significant if *p* < 0.05.

## 3. Results

### 3.1. Bacteriological Testing of Milk Samples

**Figure 1:**
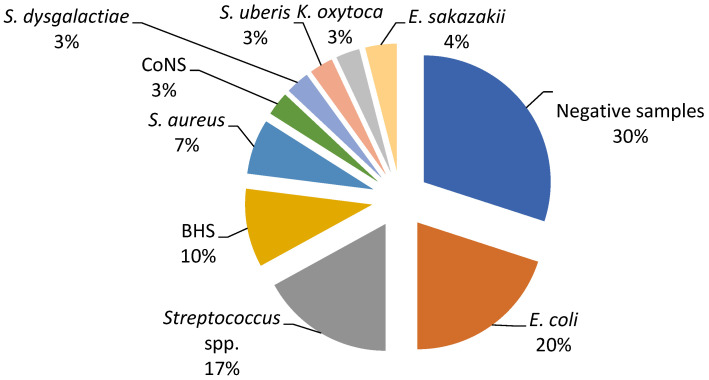
Percentage ratio of bacterial strains in the collected milk samples on Farm A.

**Figure 2:**
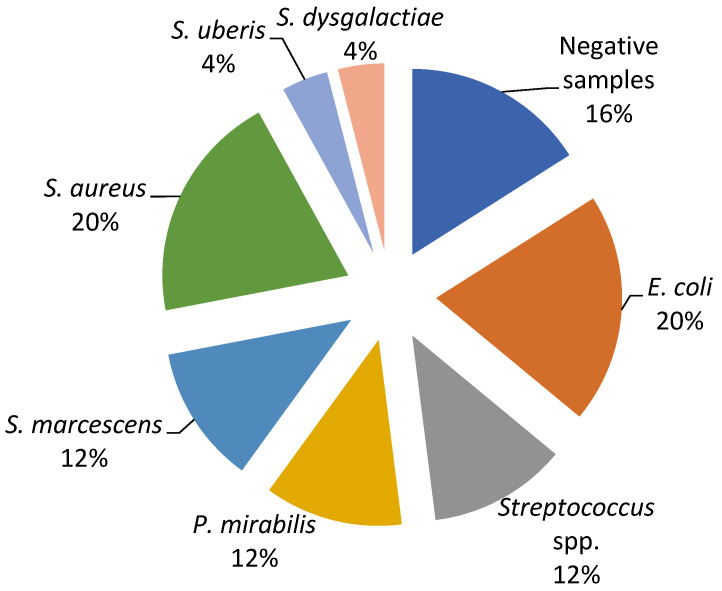
Percentage ratio of bacterial strains in the collected milk samples on Farm B.

The current study revealed a predominance of *Escherichia coli*, *Streptococcus* spp., and *Staphylococcus* spp. as the causative agents of bovine mastitis. The different isolates on Farm A included *E. coli* (20%), *Streptococcus* spp. (17%), Streptococcus beta haemoliticus (BHS) (10%), *Staphylococcus aureus* (7%), and *Enterobacter sakazakii* (4%), while Staphylococcus coagulase negative (CoNS), *Streptococcus dysgalactiae*, *Streptococcus uberis*, and Klebsiella oxytoca had a prevalence of 3% (Figure 1). The most dominant agents on Farm B were *E. coli* and *S. aureus* (20%), followed by Serratia marcescens, Proteus mirabilis, and *Streptococcus* spp. (12%), while *S. dysgalactiae* and *S. uberis* were isolated in one sample (Figure 2).

### 3.2. Antimicrobial Activity of EO-Based Pharmaceutical Formulation

The minimum inhibitory concentrations (MICs) and minimal bactericidal concentrations (MBCs) of EO-based formulations against mastitis-associated pathogens are presented in Table 1. The EO-based formulation exhibited antimicrobial activity against the tested mastitis-associated bacteria. The MIC of the formulation for the tested bacterial species ranged from 22.72 mg/mL to 45.4 mg/mL, while the lowest MIC values were found for *E. coli*, *Streptococcus* spp., and *Staphylococcus* spp. strains. The MBCs determined for the EO-based formulation ranged from 45.4 mg/mL to 90.09 mg/mL.

### 3.3. Withdrawal Period of EO-Based Intramammary Formulation of Treated Cows

#### 3.3.1. Method Validation

**Figure 3:**
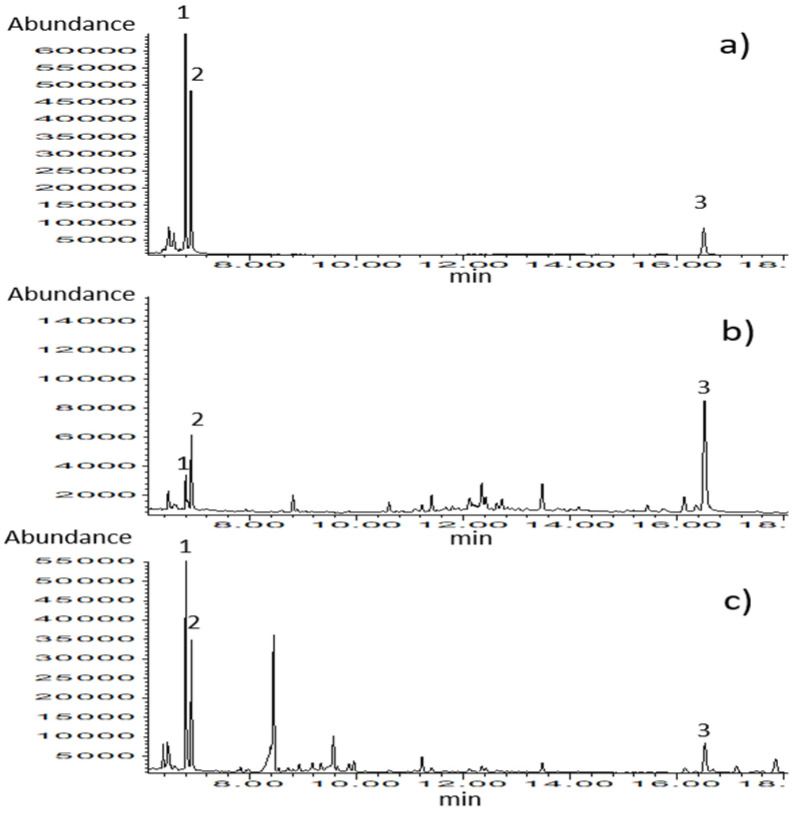
GC–MS chromatograms obtained in SIM mode for (**a**) calibration standard solution (c = 0.67 µg/mL), (**b**) blood plasma sample, and (**c**) milk sample. Identified compounds: 1-thymol, 2-carvacrol, and 3-ketamine (internal standard).

The analytical method for the simultaneous determination of thymol and carvacrol in biological matrices such as milk and blood plasma was set up and validated. Chromatograms of calibration standards (Figure 3) and samples belonging to different types of matrices confirm the selectivity of the applied analytical method. The results of the method validation are presented in Table 2.

#### 3.3.2. Thymol and Carvacrol Quantification

**Figure 4:**
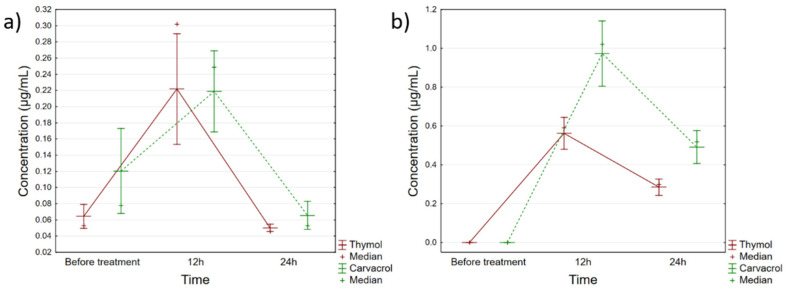
Fluctuations of thymol and carvacrol concentrations in (**a**) milk and (**b**) blood plasma of cows treated with Phyto-Bomat. Vertical bars denote 95% confidence interval.

The quantified amounts of thymol and carvacrol in the applied Phyto-Bomat preparation were 12.58 ± 1.23 mg/mL and 23.11 ± 2.31 mg/mL, respectively. Furthermore, the results of the thymol and carvacrol quantification in evaluated biological samples are presented in Supplementary Table S1, while Figure 4 shows the trends of these monoterpenes’ accumulation in milk and blood plasma.

Based on these data, it can be seen that both compounds peaked within the first 12 h after IMM dosing with Phyto-Bomat, and then declined relatively rapidly in plasma and milk (Figure 4). Regarding milk samples, there were statistically significant differences in the concentrations of thymol (F(2, 42) = 25.547, *p* = 0.000) and carvacrol (F(2, 42) = 14.882, *p* = 0.000) during the evaluated time points, whereas the post-hoc analysis indicated that the levels in samples collected 12 h after the treatment were the cause of these recorded differences. Furthermore, within about 24 h for both compounds, the same levels as before treatment were obtained. Similarly, thymol (F(2, 42) = 129.35, *p* = 0.000) and carvacrol (F(2, 42) = 92.655, *p* = *0*.000) concentrations fluctuated in the plasma, with the exception that in the plasma samples, even after 24 h certain levels were still detectable.

## 4. Discussion

Even though the treatment of bovine mastitis still relies on the use of antibiotics, both for prophylaxis and therapy, their use is questioned because of an increase in the number of resistant strains as well as residues of antibiotics in milk for human consumption [7,37,38]. Aiming to solve the problem of antibiotic resistance in bacteria, many attempts have been made to investigate the EOs’ effectiveness against mastitis-associated bacteria. Furthermore, since antimicrobial resistance poses a major threat to public health worldwide, issues related to antimicrobial use in dairy production systems are currently in focus. This implies that research should focus on the development of innovative, alternative approaches, such as using EOs. The therapeutic effects of EOs have been addressed in in vivo [13,33,39] as well as in vitro studies [4,34,40,41,42] evaluating the antimicrobial efficacy of EOs against a vast number of mastitis-associated pathogens in dairy cows.

In order to evaluate the in vitro antimicrobial efficacy of the proposed EO-based formulation (Phyto-Bomat) we have isolated the causative agents of bovine mastitis on two dairy farms where *E. coli* was the most prevalent (20%). This is in agreement with other research results [22,43,44] since *E. coli* are the most frequently isolated bacteria belonging to dairy farms with intensive systems of milk production [37]. In addition, many researchers described *Streptococcus* spp. strains as the major or minor bovine mastitis-associated pathogens worldwide [37,45]. Our research results show a high prevalence of these bacteria on Farm A and Farm B at 17% and 12%, respectively.

The analysis of EOs’ compositions is essential to confirm the presence and concentration of the active compounds responsible for EOs’ properties [46]. During the evaluation of the chemical composition and the antimicrobial activity against the most common mastitis pathogens of the EOs of oregano, mountain savory, and common and wild thyme, it was determined that carvacrol and thymol were the most abundant compounds and principally responsible for biological activity in these studies [4,34,46,47].

In the present study, the in vitro antibacterial activity of the proposed formulation (Phyto-Bomat) was tested. The results of the MIC and MBC indicate that the formulation has the highest antibacterial activity against Gram-positive strains. This finding is consistent with the literature data, where it is stated that Gram-negative bacteria have a lower susceptibility to EOs in comparison to Gram-positive bacteria [48,49]. The lower susceptibility of Gram-negative bacteria is explained by the difference in the cell wall structure, which limits the diffusion of hydrophobic compounds through the lipopolysaccharide envelope [16]. Comparing the obtained MIC values from the mixture, it is evident that higher concentrations were required to inhibit *P. mirabilis, S. marcescens, S. uberis*, and *K. oxytoca* isolates. Moreover, the results obtained in the present study are in accordance with our previous research results. Actually, the EO mixture has strong antibacterial activity in vitro, as do individual EOs obtained from common and wild thyme, oregano, and mountain savory [4,34,42].

When it comes to the proposed pharmaceutical EO-based formulation chemical composition, thymol and carvacrol were the most abundant compounds at 12.58 ± 1.23 mg/mL and 23.11 ± 2.31 mg/mL, respectively. Hence, the high antibacterial activity of the proposed formulation could be due to the high content of these compounds [27,46]. Thymol and carvacrol are known to be particularly active against microorganisms because of their phenolic structure, which can disrupt the cell membrane of microorganisms [50]. Both carvacrol and thymol are structural isomers that differ in the position of the hydroxyl group on the phenolic ring. The addition of the hydroxyl group makes them more hydrophilic, which could cause them to degrade and dissolve in microbial membranes [49]. Moreover, compared to carvacrol, thymol has a similar antimicrobial activity, even though its hydroxyl group is located in a different position. In addition, similar to carvacrol, thymol’s antimicrobial activity causes changes in the cytoplasmic membrane’s structure and function, which can harm the outer and inner membranes. It can also interact with intracellular targets and membrane proteins. Thymol’s interaction with the membrane alters the membrane permeability and causes the release of ATP and K+ ions [51,52]. Thymol integrates within the lipid bilayer’s polar head groups, inducing cell membrane alterations. In contrast to the efficiency of monoterpenes with added oxygen molecules carvacrol and thymol, monoterpene hydrocarbons p-cymene and γ-terpinene used separately do not show a remarkable inhibitory effect against bacteria [53,54].

Some blends of EOs showed more remarkable effectiveness than the single oils, highlighting a synergistic effect in relation to the phytocomplex [23]. Different types of components in the combination may affect multiple biochemical processes in the bacteria, improve the bioavailability of the combined agents, overpower the drug resistance mechanisms of bacteria, and neutralize the adverse effects of the components [55]. Moreover, some studies demonstrated stronger antimicrobial activities of EO mixtures, as compared to when they were used alone [56,57]. In most of the studies, the evaluation of carvacrol–thymol combinations showed an additive effect expressed through fraction inhibition concentration [54,58,59].

The presence of antimicrobial residues in milk is one of the biggest challenges of the food and veterinary industries worldwide since they could interfere with the production of dairy products and may cause hypersensitivity and resistance to microorganisms in humans [11]. For this reason, appropriate scientific data about how long residues remain in edible animal products are needed in order to obtain safe products of animal origin [60].

To the best of our knowledge, this is the first study where the withdrawal period of an EO-based pharmaceutical formulation in bovine mastitis treatment is studied. Withdrawal periods must be determined by studying residue depletion for a veterinary medicinal product when the target species is a food-producing animal [33]. It is expected that the compounds most abundant in plant species can be found in milk as well as in meat.

Although EOs are considered safe for human and animal consumption, negative effects linked to their use are still possible. In particular, EOs could confer an undesirable odor or taste to milk or dairy products because of their low threshold of detection [50]. Some products are already used in organic dairy cattle, but so far no scientifically based data on the withdrawal time of these plant extracts are present in the literature.

In our assay, two major chemicals (thymol and carvacrol) were identified in the milk and blood plasma of treated animals. Our research results show that administration of the proposed formulation (Phyto-Bomat) results in minimal milk residues of thymol and carvacrol, which, after 24 h return to the same level as before application. In the study conducted by McPhee et al. [33], blood and milk samples from dairy goats were analyzed for thymol residues after intramammary injections of an EO-based formulation. Residues of thymol in milk samples were only detectable 12 h post-infusion, while in plasma, thymol was detectable 15 min post-treatment up to 4 h post-infusion. On the other hand, in our study, thymol and carvacrol were detectable in plasma samples even after 24 h post-treatment. It should be taken into account that different amounts of thymol and carvacrol are present in the formulation proposed in our research and in the formulation given by McPhee et al. [33].

In general, substantial research work is needed to assess the efficacy, safety, and benefit–risk ratio of the proposed phytotherapy. It is essential to acquire data on residues, in particular when assessing consumer safety.

## 5. Conclusions

Globally, the problem of escalating microorganism resistance to the currently available antimicrobials has opened up the need for new research to find more potent treatments with a broad range of activity. Our results have demonstrated that the tested mixture of EOs exhibited antimicrobial potential against the most frequent mastitis-associated pathogens. It can also be concluded that the activity was more pronounced against Gram-positive bacteria than Gram-negative bacteria. This research should help to clarify the application of these EOs for the treatment of mastitis in the future.

Quantifying thymol and carvacrol residues in the plasma and milk of cows treated with the proposed formulation provided valuable information in terms of food safety issues. Hence, our further research results will be focused on testing the in vivo antimicrobial efficiency and clinical efficiency of the proposed EO-based formulation.

## Figures and Tables

**Table 1:** Minimum inhibitory concentrations (MICs) and minimal bactericidal concentrations (MBCs) of EO-based formulation against mastitis-associated pathogens.

Bacterial Strains	MIC (mg/mL)	MBC (mg/mL)
*Streptococcus* spp.	22.72	45.4
*Proteus mirabilis*	45.4	90.09
*Escherichia coli*	22.72	45.4
*Staphylococcus* spp.	22.72	45.4
*Serattia marcescens*	45.4	90.09
*Klebsiella* spp.	45.4	90.09
*Streptococcus uberis*	45.4	90.09

**Table 2:** Results of analytical method validation.

Analyte	Equation	R^2^	Matrix	Precision (RSD (%))		Recovery (%)	LOD (µg/mL)	LOQ (µg/mL)	Linearity Range (µg/mL)	U * (%)
				Intra-Day	Inter-Day					
Thymol	y = 5.5268x − 0.3673	0.9996	Milk	1.1	5.1	94–103	0.01	0.03	0.03–3.3	9
			Plasma	2.3	4.2	97–104	0.025	0.1	0.1–10	8
Carvacrol	y = 4.3277x − 0.2438	0.9995	Milk	0.8	4.7	97–104	0.03	0.05	0.03–3.3	10
			Plasma	1.3	4.3	96–105	0.1	0.3	0.1–10	9

* U-expanded measuring uncertainty (k = 2).

## Data Availability

The data used to support the findings of this study are available in the present manuscript.

## Supplementary Materials

The following supporting information can be downloaded at: https://www.mdpi.com/article/10.3390/ijerph192416643/s1, Table S1: Quantification of thymol and carvacrol in milk and blood plasma.
